# Separability of Lexical and Morphological Knowledge: Evidence from Language Minority Children

**DOI:** 10.3389/fpsyg.2018.00163

**Published:** 2018-02-21

**Authors:** Daphna Shahar-Yames, Zohar Eviatar, Anat Prior

**Affiliations:** ^1^Edmond J. Safra Brain Research Center for the Study of Learning Disabilities, University of Haifa, Haifa, Israel; ^2^Department of Learning Disabilities, Faculty of Education, University of Haifa, Haifa, Israel; ^3^Department of Psychology, University of Haifa, Haifa, Israel

**Keywords:** vocabulary, lexical knowledge, morphology, language-minority, bilingual, type, token

## Abstract

Lexical and morphological knowledge of school-aged children are correlated with each other, and are often difficult to distinguish. One reason for this might be that many tasks currently used to assess morphological knowledge require children to inflect or derive real words in the language, thus recruiting their vocabulary knowledge. The current study investigated the possible separability of lexical and morphological knowledge using two complementary approaches. First, we examined the correlations between vocabulary and four morphological tasks tapping different aspects of morphological processing and awareness, and using either real-word or pseudo-word stimuli. Thus, we tested the hypothesis that different morphological tasks recruit lexical knowledge to various degrees. Second, we compared the Hebrew vocabulary and morphological knowledge of 5th grade language minority speaking children to that of their native speaking peers. This comparison allows us to ask whether reduced exposure to the societal language might differentially influence vocabulary and morphological knowledge. The results demonstrate that indeed different morphological tasks rely on lexical knowledge to varying degrees. In addition, language minority students had significantly lower performance in vocabulary and in morphological tasks that recruited vocabulary knowledge to a greater extent. In contrast, both groups performed similarly in abstract morphological tasks with a lower vocabulary load. These results demonstrate that lexical and morphological knowledge may rely on partially separable learning mechanisms, and highlight the importance of distinguishing between these two linguistic components.

## Introduction

Children learn the language used around them through their everyday social interactions and experiences. This exposure enables them to learn both specific lexical items as well as abstract linguistic regularities. Such abstract knowledge about language patterns in turn enables children to understand new words and construct sentences they have never heard before (Tomasello, 2003). Thus, Usage-Based models posit that the acquisition of linguistic knowledge is input-driven and is influenced by the frequency with which children encounter linguistic forms in the language they hear (for alternative, generative, approaches to language acquisition, see Chomsky, 1980; Pinker, 2015). Two aspects of frequency are important: *Token* frequency is the amount of exposure to a specific language unit, for example a word. *Type* frequency is the exposure to language regularities, whether they are syntactic, phonological or morphological. Type frequency, therefore, counts the number of times a child is exposed to a specific pattern in a given language (Bybee, 1995, 2007; Tomasello, 2003). As such, token frequency is a strong driver of vocabulary knowledge, whereas type frequency influences the acquisition of regularities, including morphological structures. Repeated exposure to, and use of, individual tokens leads to the accumulation of a “critical mass” of exposure to their shared type, or regularity (Marchman and Bates, 1994; Nicoladis et al., 2007). This critical mass enables the child to generalize about morphological patterns by making associations among related words from his/her lexicon (Bybee, 1995; Tomasello, 2003).

Critically, however, for a given learner, token and type frequency are linked to each other—with greater amounts of linguistic input, the learner is exposed both to more individual tokens, and to more numerous examples of given types. Thus, educational research among school-aged children has found that the acquisition of lexical and morphological knowledge are positively related (Nagy and Anderson, 1984; Ku and Anderson, 2003; McBride-Chang et al., 2005; Sparks and Deacon, 2015; Goodwin et al., 2017), a relation that holds in longitudinal studies as well (McBride-Chang et al., 2005, 2008; Sparks and Deacon, 2015). Structural equation modeling has also demonstrated that morphological knowledge as a general factor had strong associations with vocabulary (Goodwin et al., 2017). Finally, an intervention study among kindergarten children implementing combined instruction of vocabulary and morphological awareness, showed reciprocal associations between vocabulary and morphological skills. Thus, gains in morphological awareness were associated with higher initial levels of vocabulary and independently with gains in vocabulary and also gains in vocabulary were associated with higher initial levels of morphological awareness and, independently, with gains in morphological awareness (Ramirez et al., 2014).

This association between morphology and lexical knowledge is most likely driven not only by variability in the amount of language exposure among children, but also by reciprocal influences between learning words and learning patterns. Spencer et al. (2015) suggested that morphological knowledge is an integral part of vocabulary knowledge. Carlisle (2007) on the other hand, proposed that morphological knowledge drives vocabulary development, as children find it easier to learn new words that conform to familiar morphological patterns. Combining these two views, McBride-Chang et al. (2008) as well as Ramirez et al. (2014) suggested bidirectional connections between the two abilities, such that knowledge of morphemes aids vocabulary development, and in turn, a large vocabulary supports the development of morphological awareness.

Although the acquisition of vocabulary and morphology are both input driven, it is important to note that they also recruit distinct learning mechanisms. Whereas acquiring vocabulary requires associative learning and linking phonological forms to semantics, the acquisition of morphology relies on extracting regularities in the input (Clark, 1998; Lignos and Yang, 2018). Indeed, previous research has demonstrated sensitivity to morphological regularities even in pseudo-word stimuli (Deutsch et al., 1998; Berent et al., 2004, 2007). Thus, it should be theoretically possible to find dissociations between lexical and morphological knowledge in different learners. Specifically, variability in the amount and composition of language exposure might have different consequences for lexical and morphological knowledge. In the current study, we investigate this possibility by comparing language minority children, who have reduced exposure to the societal language, with their native speaking peers and examine the consequences of this reduced exposure for both lexical and morphological knowledge.

In addition, it is possible that some of the previously reported correlations between vocabulary and morphology were at least partially driven by the tools used to assess morphological knowledge. Many previous studies examining this link assessed morphological knowledge by requesting children to produce or judge real word stimuli (e.g., Kieffer and Lesaux, 2012a; Sparks and Deacon, 2015; Goodwin et al., 2017). We argue that such tasks by necessity also recruit children's knowledge of specific lexical items, and are therefore, not ideally suited to examine abstract morphological knowledge independently. In the current study, we investigate this possibility by administering a battery of four morphological tasks (see below) and directly assessing their correlation with vocabulary, as reflecting the degree to which they recruit lexical knowledge.

The current study, therefore, investigates the possible separability of lexical and morphological knowledge using two complementary approaches. First, the study includes several measures of morphological knowledge, using either real-word or pseudo-word stimuli, to ask whether such task parameters indeed are differentially associated with vocabulary knowledge. Second, as described above, we tested language minority children, who have reduced exposure to the societal language, to ask whether such reduced exposure has parallel impact on both aspects of linguistic knowledge.

### The complexity of measuring morphology

In contrast to vocabulary, which is a well-defined construct with established and standardized measures, there is large variability in the definition and evaluation of morphological abilities (Goodwin et al., 2012, 2017; Tighe and Schatschneider, 2015). In the current study, we use the general term “morphological knowledge” (Bowers et al., 2010; Nagy et al., 2014). This term includes both morphological awareness, which refers to “awareness of the morphemic structure of words and the ability to reflect on and manipulate that structure” (Carlisle and Feldman, 1995, p. 194) and morphological processing, which is a less conscious use of morphological information (Bowers et al., 2010; Nagy et al., 2014). Notably, it is not always easy to draw the boundary between morphological awareness and processing, because a given measure of performance for an individual child may rely to different extents on strategic and less conscious access to morphology.

In practice, *morphological awareness* is often measured by using cloze tasks (Carlisle, 2000; Ramirez et al., 2011; Apel et al., 2013; Kraut, 2015) or word analogy tasks (Kirby et al., 2012; Sparks and Deacon, 2015). For example, the experimenter provides the participant with a probe base word, and the child is then requested to use an inflected or derived form of the word to complete a sentence [e.g., the target word **magic** is presented and then the sentence: The performer was a good _____ (magician)].

On the other hand, tasks designed to investigate *morphological processing* attempt to assess to what degree participants rely on morphological knowledge in performing a given task. For example, priming paradigms measure the degree of facilitation in processing target words when they are preceded by a morphologically related prime (e.g., Frost et al., 2013; Kraut, 2015). Studies conducted among adults using masked priming and cross-modal priming paradigms, show morphological priming effects that cannot be reduced to phonological or semantic overlap in word recognition and lexical decision tasks (e.g., Frost et al., 1997, 2000). Morphological priming effects have also been demonstrated in children in various languages (Quémart et al., 2011; Beyersmann et al., 2012; Shalhoub-Awwad and Leikin, 2016), including Hebrew (Schiff et al., 2012). However, we could not identify studies using the priming paradigm among language minority children.

Importantly, many of the studies examining morphological knowledge in school-aged children, have used both real words and pseudo-words as stimuli, without necessarily directly comparing them (McBride-Chang et al., 2005; Siegel, 2008; Goodwin et al., 2012; Kieffer and Lesaux, 2012a; Park et al., 2014; Sparks and Deacon, 2015; Spencer et al., 2015). Performance on such tasks, therefore, might reflect both morphological and lexical knowledge, and does not allow a clear distinction between the two (McBride-Chang et al., 2005; Kuo and Anderson, 2006; Tighe and Schatschneider, 2015). A second issue is that some studies have examined morphological knowledge using irregular or exception words (e.g., Kirby et al., 2012). From the perspective of questioning the separability of morphological and lexical knowledge this is problematic, because the learning of such items relies on token frequency (and not on type frequency), and thus would arguably lead to higher estimates of the relation of morphological knowledge to lexical knowledge.

In the current study, we examine this issue by including tasks probing the knowledge of abstract morphological regularities by using pseudo-word stimuli, and directly comparing them with tasks using real-word stimuli. Thus, we can examine whether morphological tasks including pseudo-word stimuli have weaker links with lexical knowledge than real-word tasks, allowing for a clearer distinction between type- and token-frequency based learning.

### Language minority students—the impact of reduced exposure on morphological and lexical knowledge

Language minority students speak a home language that differs from the societal language and thus have lower exposure to the societal language (for reviews see, August and Shanahan, 2006; Geva and Wiener, 2015). Differences in the amount of children's exposure to, and use of, a particular language can influence their vocabulary acquisition. Indeed, there are well-documented and persistent gaps in vocabulary between native speakers and language minority children (e.g., Geva, 2006; Jean and Geva, 2009; Schwartz et al., 2009; Kieffer and Lesaux, 2012b). Such a gap demonstrates that even after a number of years of instruction in the societal language, language minority speakers do not catch up with their native speaking peers in terms of vocabulary knowledge (Farnia and Geva, 2011; Mancilla-Martinez and Lesaux, 2011; Kieffer and Lesaux, 2012b).

These gaps in vocabulary knowledge have been attributed to language minority children's reduced exposure to the society language, and thus lower token frequency of specific lexical items. However, the question still remains regarding the impact of reduced language exposure on school-aged language minority children's accumulation of *type* frequency. It is possible that by this age, despite their reduced exposure to the society language, language minority children have nonetheless accumulated sufficient type exposure, and have successfully extracted morphological regularities on par with their native speaking peers. Alternatively, it might be the case that language minority children have not yet achieved the necessary “critical mass” and will show persistent gaps in abstract morphological knowledge when compared with native speaking peers, due to their reduced type frequency exposure (Tomasello, 2003; Gathercole, 2007; Nicoladis et al., 2007).

Further complicating this issue is the fact that morphological knowledge can be tested using either regular or irregular items—as has often been the case, for example, in studies focusing on past-tense formation in English (e.g., Bybee and Slobin, 1982). This distinction is of special importance in the current context, because knowledge of irregular forms relies more on the frequency of exposure to these specific tokens, whereas knowledge of regular forms relies on frequency of type exposure.

Reduced token frequency of specific items is linked, as mentioned above, to smaller vocabulary and also to reduced acquisition of morphologically irregular words. Indeed, there is a substantial body of knowledge regarding the acquisition of specific morphological structures in young children acquiring a second language (e.g., Nicoladis et al., 2007; Schwartz et al., 2009). Most of these studies target Germanic (e.g., English: Blom et al., 2012; Dutch: Rispens and de Bree, 2015; German: Schönenberger et al., 2012) or Latinate (e.g., French: Nicoladis and Paradis, 2012) languages, and children before elementary school age. These studies mostly report reduced morphological knowledge of bilingual children relative to that of monolingual counterparts, especially in irregular forms (Nicoladis et al., 2007; Paradis et al., 2010; Rispens and de Bree, 2015). As noted above, learning of irregular forms relies mostly on exposure to the specific tokens, and thus is less informative regarding the impact of reduced exposure on language minority children's ability to extract regularities based on type frequency.

Evidence regarding the morphological knowledge of school-aged language minority children is less conclusive, in studies using both real word and pseudo-word stimuli. Some studies have found language minority learners to have lower morphological knowledge than native speaking peers (Goodwin et al., 2012; Kieffer and Lesaux, 2012b; Kieffer and Box, 2013). In contrast, other studies found no differences in English morphological knowledge among 6th grade native English and language minority typical readers (Siegel, 2008) and struggling readers (Lesaux and Kieffer, 2010). Finally, Ramirez et al. (2011) found differences between language minority and native speaking children on some aspects of morphological knowledge, depending on the language minority students' L1.

In light of the well-established findings of language minority students' gaps in vocabulary, it is especially important to distinguish between morphological and lexical knowledge when investigating this population. Otherwise, findings of reduced morphological abilities in language minority children might actually reflect their smaller vocabularies, and not necessarily differences in their representation of and access to morphological paradigms. However, the studies reviewed above do not distinguish between real word and pseudo-word morphological tasks, and thus are less informative in this regard.

To enable a better distinction between lexical and morphological knowledge, the current study specifically compares morphological tasks using pseudo-word stimuli with more common tasks using real words. In addition, the current study examines knowledge of Hebrew, a language that has a rich and salient morphological structure, and thus can putatively lead to a more nuanced investigation of morphological knowledge.

### Hebrew morphology

Hebrew is a Semitic language with a complex morphological structure. Most Hebrew words are constructed from a root and a pattern. The root, which usually consists of three consonants, carries the main semantic meaning, whereas the word pattern carries mostly grammatical derivational information. These basic morphemes are abstract and only the combination of the consonantal root and the vocalic pattern creates meaningful words (Frost, 2009; Berman, 2016). For example, the word *zimra* (singing) is a derivation of the root *z.m.r* and the pattern CiCCa. Thus, Hebrew word learning is dependent on the connections of the word with morphologically related word families (Ravid, 2011; Berman, 2016).

Hebrew relies heavily on inflectional and derivational morphology, both of which have a protracted developmental trajectory from early childhood through adolescence (for a recent review see, Berman, 2016). In the current study, we chose to focus on derivational morphological knowledge because whereas the bulk of inflectional knowledge is early acquired, a substantial amount of derivational knowledge is established only during the school years. Thus, investigating derivational morphological knowledge is more appropriate in light of the current age group, of upper elementary school children (Ravid and Schiff, 2006; Ben-Zvi and Levie, 2016).

In the current study, we examine the morphological knowledge of language minority students, who accumulate lower levels of exposure to various morphological pattern types. Such reduced type exposure might cause less difficulty in languages, like English, with a small number of morphological inflections and derivations. For example, past tense formation in English requires exposure to one regular inflection pattern, namely—ed (which covers half of the 60 earliest acquired verbs for English-speakers) (Bybee and Slobin, 1982). Therefore, when children need to inflect a low-token frequency item, they can mostly do so successfully by direct comparison to other forms. In English, irregular forms, which constitute a high percentage of the young lexicon, are varied in form and have to be learned individually, and are thus reliant on token exposure (Bybee, 1995; Nicoladis et al., 2007).

However, the distribution of morphological patterns in Hebrew is dramatically different (Schwarzwald, 2001), because the verb paradigm includes seven verb-patterns (*Binyanim*), used for verb derivation from roots to indicate for example, active/passive, reflexivity etc. (see further description in Schwarzwald, 2001). Even the inflectional system is more variable, as for example the regular past tense inflection has eight forms, depending on person, number and gender. However, in contrast to English, there is a much lower percent of irregular forms, both in derivation and in inflection. Thus, the morphological variability in Hebrew lies within the complex regular paradigms and is not a result of a simple rule with many exceptions.

Previous studies of Russian-Hebrew language minority children, which are the targeted population of the current study, investigated only inflectional morphology. Two studies investigating children aged 3–8 found no differences between language minority and native Hebrew speaking children in producing pluralization of regular forms (type exposure) but showed some disadvantage for the language minority children in producing irregular plural forms (Token frequency exposure) (Schwartz et al., 2009, 2014). A different pattern emerged in a study investigating the inflectional abilities of native Russian speaking college students, who had immigrated to Israel as adolescents. This study shows lower performance of Russian-Hebrew students across both regular and irregular inflectional categories examined in the study (Alfi-Shabtay and Ravid, 2012), suggesting that reduced type exposure might lead to gaps in learning the morphological paradigm itself. The current study expands on these somewhat mixed findings, by further investigating the impact of the reduced exposure on vocabulary and derivational morphology in the context of Hebrew, a language with a complex morphological paradigm.

### The current study

The current study investigated the possible separability of lexical and morphological knowledge using two complementary approaches. First, we administered four distinct tasks measuring different aspects of morphological knowledge, both awareness and processing, to test the hypothesis that different morphological tasks recruit lexical knowledge to various degrees. To this end, two morphological tasks required children to manipulate Hebrew pseudo-words. Performance of these tasks recruits abstract morphological knowledge and is putatively less dependent on lexical knowledge. The two additional morphological tasks required children to process real Hebrew words, thus arguably recruiting lexical knowledge to a greater extent. In addition to testing the central hypothesis, two of the tasks (one pseudo-word and one real-word tasks) target morphological processing, whereas most previous research has targeted morphological awareness exclusively. This was motivated by our broad perspective on morphological knowledge.

Second, we compared the vocabulary and morphological knowledge of 5th grade Russian minority speaking children in Israel, where the societal language is Hebrew, to that of their native speaking peers. This comparison allows us to ask whether reduced exposure might differentially influence lexical and morphological knowledge.

Specifically, the current study seeks to answer the central question regarding the possible independence of acquiring lexical and morphological knowledge, and whether these types of knowledge might rely on at least partially distinct learning mechanisms. Such distinct mechanisms would be supported if the following patterns are evident in the results: First, morphological measures using pseudo-words would be less strongly associated with vocabulary knowledge, for all children. Second, the reduced exposure of language minority learners to Hebrew will lead to reduced vocabulary knowledge, but mostly intact morphological knowledge, especially in measures using pseudo-word stimuli. This is because their exposure has been sufficient to reach a “critical mass” that has allowed them to generalize abstract morphological knowledge equal to that of native speaking peers (Marchman and Bates, 1994; Gathercole, 2007). Finally, it is more difficult to put forth a strong hypothesis regarding the distinction between morphological awareness and processing, because this dimension has received less attention in the literature on language minority children. We can tentatively suggest that language minority students might show smaller differences from native speaking children in morphological awareness than in morphological processing tasks, due to previous reports in the literature of enhanced meta-linguistic (though not specifically morphological) awareness in this population (Bialystok, 2001).

This novel investigation has the potential of expanding our understanding of the complex relations between lexical and morphological knowledge, by using various morphological tasks and examining language minority learners, in a context of a morphologically complex language other than English.

## Methods

### Participants

Participants were 114 5th grade students from five different public elementary schools from an urban area in the north of Israel. The sample was drawn from regular classes, such that students are typically developing with no sensory-motor difficulties. Fifty-six students (52% girls) reported speaking Hebrew exclusively at home and were classified as native Hebrew speakers, and 58 students (65% girls) reported Russian as their native language and were classified as Russian-speaking minority learners. The two language groups are a result of convenience sampling, yet all participants were drawn from the same classrooms, from schools in similar neighborhoods with equivalent middle-low socio-economic status (see sample characteristics in Table 1).

**Table 1:** Participant characteristics.

	**Native Hebrew speaking students (*n* = 56)**	**Language minority students (*n* = 56)**
Age (years; months)	11;02 (0.33)	11;05 (0.46)
Toni III (Non-verbal ability test)	25.09 (7.15)	23.39 (7.61)
**PARENTAL EDUCATION (YEARS)**		
Mother	14.18 (2.50)	13.40 (2.30)
Father	13.80 (2.19)	13.04 (2.65)
**PARENT SELF-RATED LANGUAGE PROFICIENCY (0–5)**		
Mother's Hebrew prof.^**^	4.87 (0.33)	2.96 (1.43)
Mother's Russian prof.^**^	–	4.90 (0.23)
Father's Hebrew prof.^**^	4.79 (0.43)	2.58 (1.36)
Father's Russian prof.^**^	–	4.48 (0.91)

Parent questionnaires included a self-rating language proficiency scale between 0 (no proficiency) to 5 (very proficient) in oral, reading and writing skills in Hebrew and Russian. The average score was calculated across all skills in each language.

** *p < 0.001*.

In order to identify suitable participants, letters describing the study and seeking parental approval were distributed to all 5th grade students from participating schools. The letter included basic questions about home language environment and parental self-ratings of Hebrew and Russian oral proficiency and literacy. At this stage, children who spoke languages other than Hebrew and Russian at home were excluded from the study. Children whose parents approved participation were divided into two groups.

A majority of the of the language minority students were second-generation immigrants, as 78.5% were born in Israel. One child had one Russian speaking parent and one Hebrew speaking parent, and can be considered to be a simultaneous bilingual with exposure to both languages from birth. All other children came from families in which both parents had emigrated from the Former Soviet Union countries, or from single-parent families. According to parental reports, 64% of these sequential bilinguals were exposed to Hebrew from age 2 to 3 years. The entire sample had attended Hebrew speaking public schools from age six, namely the first grade.

All language minority students reported speaking Russian at home on a regular basis—half reported speaking Russian exclusively with their parents and the rest spoke both Russian and Hebrew at home. Russian language proficiency was also assessed objectively using a Russian receptive vocabulary test administered by a native Russian speaker (Schwartz, 2006). The average score of the language minority group in Russian was 76 correct items, out of 110 (*SD* 13.54). Thus, although the language minority students are mostly second-generation immigrants and Hebrew is the only instructional language at school, the participants have significant oral language abilities in Russian^1^. Finally, two students initially identified as belonging to the language minority group, but who scored more than two standard deviations below the mean of the group on the vocabulary measure were excluded from the sample, leading to a final group of 56 language minority students.

### Measures

#### Background measures

##### Non-verbal ability

Non-verbal ability was measured to match groups on this background variable using the Test of Nonverbal Intelligence-3 (Brown et al., 1982). The test includes five training items and 45 abstract/figural problem-solving items arranged in increasing order of difficulty. Items are in multiple-choice format, with either four or six options. Participants selected and marked the best option. Internal consistency for the original test is reported as between 0.8 and 0.9 (Brown et al., 1982).

##### Phonological awareness

Phonological awareness was assessed using a subtest of the standardized Alef Ad Taf test (Shany et al., 2006). This subtest measured children's ability to segment 16 spoken words into phonemes, by asking the participants to omit a phoneme from a real-word. Percent of correct responses was calculated. Internal consistency reported for the original test (α Cronbach) is 0.87 in fourth grade (Shany et al., 2006).

##### Productive vocabulary

Hebrew vocabulary knowledge was assessed using a picture naming test (Kavé, 2006) consisting of 48 black-and-white line drawings, each depicting an object corresponding to a Hebrew noun, presented according to descending word frequency. Participants were instructed to name each picture using one word, and the number of correct answers was calculated. Standardized scores are available for Hebrew native speaking children (Kavé, 2006). Split half reliability reported for the original test is 0.6 (Kavé, 2005).

#### Morphological knowledge

Morphological knowledge was assessed using four distinct tasks. We opted to use measures that had been used in previous studies with Hebrew speakers. In Table 2, we detail how these measures are mapped to morphological awareness and processing, and whether they utilize real word or pseudo-word stimuli.

**Table 2:** Theoretical mapping of morphological tasks.

	**Morphological awareness**	**Morphological processing**
Real word stimuli	Real word sentence completion	Cross modal priming
Pseudo-word stimuli	Pseudo-word sentence completion	Pseudo-word reading aloud

##### Tests of morphological awareness

*Real word sentence completion*. This task is an experimental task designed by Ben-Zvi and Levie (2016). Participants were presented with an oral sentence, which included a stimulus word from the same root as the expected response, but from a different morphological pattern. For example: Mom **sent** (∫*alxa*) the letter. What happened to the letter? The letter ____ (**was sent**/*ni*∫*lax*). In this example, the root ∫*.l.x* appears in the prompt sentence in the active form of the verb, with the pattern CaCCa, and the target response is the same root, ∫*.l.x*, in the passive voice, with the pattern niCCaC. Children's responses were transcribed online, and coded afterwards for accuracy.

The task included 31 sentences: six nouns, 10 adjectives and 15 verbs presented in the same preset order to all participants, by lexical category. Before each lexical category three examples were given. Performance was first scored on a scale reflecting overall accuracy (1 point for each correct answer). Average performance was calculated for each participant, and this score was used in calculating cross-task correlations. Internal consistency for this scoring method was satisfactory (Cronbach's alpha = 0.78) after eliminating one item^2^.

A second scoring scheme gave participants credit for partial knowledge. The partial knowledge score relied on a detailed analysis, with one point given for each of the following: (a) the answer included the same root as the stimulus word (Root); (b) the answer used an acceptable morphological pattern, given the context (Root+Pattern); (c) the answer is an existing word in Hebrew (Root+Pattern+Lexical) and (d) the answer is semantically acceptable in the context of the sentence (Fully correct answer). Thus, each answer could receive a score between 0 (for a completely wrong or omitted response) to 4 (for a fully correct response). This partial-knowledge scoring scheme allowed us to analyze children's response strategy when they were unfamiliar with the required lexical item, through error analysis.

*Pseudo-word sentence completion*. This task was based on an experimental task developed for Hebrew speakers (Zeltsman-Kulick et al., under review), based on the Pal-II (Berninger, 2007). Students were presented with 14 written sentences in which one word was missing, and were instructed to complete the sentence by choosing the correct pseudo-word out of four options, based on the morpho-syntactic context. An example from the English version of the task is “I like to _______,” (a) blip (b) blipingly (c) blipness (d) blipable (for the full task in Hebrew, see Appendix 1 in Supplementary Material).

Sentences were also read out loud by the experimenter. Thus, although the targets were all pseudo-words, task completion relied on correctly identifying the thematic/syntactic roles of these items, based on their morphological structure. All pseudo-words were based on the same pseudo-root (*p.k.l*) embedded in various Hebrew word patterns for verbs, adjectives and nouns. Percent of correct responses was calculated for each participant. Internal consistency in the current study (alpha Cronbach) was .70.

###### Tests of morphological processing

*Cross-modal priming*. The cross-modal priming task was used to examine whether exposure to a morphologically related prime facilitates lexical decision on a subsequent target, as a measure of reliance on morphological information in processing. Morphological priming from the root morpheme has been previously demonstrated in Hebrew speaking adults (Frost et al., 1997, 2000; Deutsch et al., 1998) and children (Schiff et al., 2012).

The cross-modal task used in the current study was adapted from Frost et al. (2000) and includes four priming conditions. In the morpho-semantic related condition, the prime is derived from the same root as the target, and is semantically related to it. In the pure morphological related condition, the prime is derived from the same root as the target, but is unrelated to it in meaning. In the phonological control condition the prime and target share the same number of letters and phonemes as in the critical related conditions, but are morphologically unrelated. In the unrelated control condition prime and target have no orthographic or phonological overlap (for a full example, see Table 3).

**Table 3:** Example of materials used in the cross-modal priming task.

	**Morpho-semantic**	**Pure morphological**	**Phonological control**	**Unrelated control**
Auditory prime	*Madrix* (a guide) 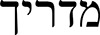	*Drixut* (alertness) 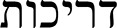	*Mehudar* (fancy) 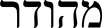	*Shlemut* (perfection) 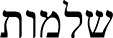
Visual target	*Hadraxa* (guidance) 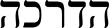			

The primes and targets of the critical conditions (morpho-semantic, pure morphological and phonological control) were taken directly from previous studies (Frost et al., 1997, 2000). The stimuli for the unrelated control condition were designed for this study, and retained the principals of the original study, namely no orthographic or phonological overlap between prime and target. This condition was included to match the current design with the previous studies (Frost et al., 1997, 2000), but results from this condition were not analyzed and not compared with the experimental conditions. In order to adapt the task for children, four words from a high linguistic register were changed to more frequent words (for example, the word “a*rik*” which means “renegade” was replaced). In addition to the 48 target words, the task also included 48 non-words to enable lexical decision. Target words were divided into four lists of 12 words each. The lists were rotated across priming conditions for different participants, such that each target appeared in each priming condition equally often, albeit for different children. Thus, the very same targets were compared across the experimental conditions, and in effect served as their own control (see also Frost et al., 2000, for similar methodology).

Children performed the cross-modal priming task on a laptop computer, wearing headphones for optimal delivery of the auditory prime. Children responded by key press, using the index finger of both hands—the “m” was used to indicate a real word and the “z” was used to indicate a non-word. These two keys on the keyboard were indicated by colored stickers. The experimental script was controlled by E-prime (Schneider et al., 2002). Each trial started with a fixation stimulus +++ presented for 1,000 ms, immediately followed by an auditory presentation of the prime. The visually presented target appeared on the computer screen at the offset of the prime. Targets remained on the screen until the participant made a lexical decision. Accuracy and reaction time were measured. Before starting this task, a practice session was carried out to ensure the participants understood the requirements and were able to perform the task. In addition, the task was divided into four blocks of 24 trials each, with brief breaks introduced between blocks. An experimenter was present while participants completed the priming task, and ensured that they were focused before beginning each block. Priming in the pure morphological and the morpho-semantic conditions was calculated by comparing them with the phonological control condition, as in the original studies (Frost et al., 1997, 2000).

*Pseudo-word reading aloud* This experimental task was designed by Bar-On and Ravid (2011) to investigate morphological processes involved in decoding unfamiliar words. Hebrew has two scripts—one that includes vowel diacritics and thus is fully phonologically specified, and a second unvowelized script, which provides partial phonological information, mostly consonants (for more detailed description see, Share and Bar-On, 2017). Children acquire literacy using the vowelized script, which is replaced by the unvowelized script during third grade. Thus, the participants in the current study had been reading the unvowelized script for at least 3 years. The pseudo-word reading aloud task capitalizes on the inherent under-specification of the unvowelized script to probe morphological processing. In order to read these unvowelized words aloud, the reader must insert vowel information. Morphological processing is demonstrated when children assimilate the pseudo-words to existing morphological patterns, rather than inserting random vowel information.

Participants were instructed to read aloud a list of 20 unvowelized pseudo-words constructed by embedding pseudo-roots in real Hebrew word patterns. For example, the consonant string HTRZF (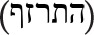) is presented, but because vowel information is not available, participants need to add such information when pronouncing the word aloud. Thus, the string HTRZF can be read as *hitrazef* based on the verbal pattern hitCaCeC, or alternatively can be read as *hetrizaf*, which does not adhere to any existing morphological pattern in Hebrew. Critically, both readings are equally plausible by the underspecified letter string presented. Thus, when reading unvowelized script without context, only morphological processing can guide readers to specific vowel patterns, and away from random vowelization (Bar-On and Ravid, 2011). Children's reading of the words was audio recorded, and then coded and scored offline. Each response that adhered to an existing morphological pattern in Hebrew received a score of 1 and all other responses received a score of 0 (for coding criteria, see Bar-On and Ravid, 2011)^3^. Internal consistency of this task in the current study (alpha Cronbach) was 0.72.

### Procedure

The current study was part of larger project, which also investigated literacy skills of language minority learners (Shahar-Yames and Prior, in press). Participants were administered a battery of tests in February through May of 5th grade, in two testing sessions, each lasting ~1 h. The two sessions were administered during the same week. One session was administered individually and included the productive vocabulary task, the real word sentence completion task, the pseudo-word reading aloud task and the computerized cross-modal priming task. The other session was administered in a group setting of 5–8 children and included the pseudo-word sentence completion task and the non-verbal intelligence task. Session order was counterbalanced across participants from both groups. The order of tasks within each session was fixed. All tasks were administered during school hours in a quiet room by the first author and trained graduate students from the Department of Learning Disabilities.

## Results

### Links between morphology and vocabulary

The relations between vocabulary and morphological knowledge were examined by exploring the correlations of the different morphological tasks with each other, with expressive vocabulary and with phonological awareness (Table 4). The pattern and magnitude of correlations were similar for the two language groups when analyzed separately (see Appendix 2 in Supplementary Material), and are therefore presented jointly for the entire sample. To correct for multiple correlations, we set the alpha level at 0.01.

**Table 4:** Correlations among morphological knowledge, vocabulary, and phonological awareness tasks for the entire sample (*N* = 112)^*^.

**Measure**	**2**	**3**	**4**	**5**	**6**
1. Real word sentence completion	0.50^**^	0.29^**^	−0.17	0.71^**^	0.45^**^
2. Pseudo-word sentence completion		0.43^**^	0.06	0.47^**^	0.47^**^
3. Pseudo-word reading aloud			0.13	0.23	0.48^**^
4. Morphological RT priming effect				−0.12	−0.07
5. Productive vocabulary					0.30^**^
6. Phonological awareness					

* p < 0.01;

** *p < 0.005. For the real-word sentence completion task, we used the overall accuracy score (not the partial knowledge score). For the morphological priming, we used the priming effect in RT—subtracting performance in the morphologically related condition from performance in the phonological control condition*.

Three out of the four morphological tasks were significantly, moderately correlated: real word sentence completion, pseudo-word sentence completion and pseudo-word reading aloud, and seem to be measuring shared abilities. Surprisingly, the morphological priming effect was not significantly correlated with the other morphological tasks, and was further not correlated with either phonological awareness or vocabulary knowledge. This pattern suggests that the priming task might be measuring somewhat different processes, or is influenced by additional mechanisms (e.g., speed of processing or decision making). We therefore delay discussion of the priming task and first discuss the three related morphological tasks.

All three morphological tasks were moderately positively correlated with phonological awareness, as has been previously reported (e.g., Nagy et al., 2006; Ramirez et al., 2011). Two of the morphological tasks were also positively correlated with productive vocabulary, but the magnitude of the correlation varied. We hypothesized that tasks using real words would correlate more strongly with vocabulary knowledge than tasks using pseudo-word stimuli. The results mostly support this hypothesis. Specifically, the strongest correlation was found between vocabulary and the real word sentence completion task (*r* = 0.71), the pseudo-word sentence completion task was moderately correlated with vocabulary (*r* = 0.47) and the pseudo-word reading aloud task was only weakly and not significantly correlated with vocabulary (*r* = 0.23). These findings indicate that indeed the real-word sentence completion task recruits lexical knowledge to a great extent. Further, the difference between the two pseudo-word tasks can be understood by the fact that in the pseudo-word sentence completion task, performance relies on meaningful linguistic context, whereas in the pseudo-word reading aloud task, there is no additional linguistic information.

The above reported pattern of correlations suggests that the three morphological tasks tap into shared variance in morphological abilities, since they are moderately correlated with each other. At the same time, the differential links between the morphological tasks and vocabulary knowledge suggest that the tasks do not recruit lexical knowledge to the same degree, supporting the notion of separability between morphological and lexical knowledge.

### Group differences in vocabulary and morphological knowledge

Vocabulary and morphological knowledge in Hebrew were compared between the language minority and the native Hebrew speaking students (see Table 5)^4^. Analyses by subject are reported as *t*_1_ and analyses by items are reported as *t*_2_.

**Table 5:** Mean accuracy (*SD*) on vocabulary, phonological awareness, and morphology tasks, by language group.

	**Native Hebrew**	**Language minority**
Vocabulary^**^	84% (7)	72% (13)
Phonological awareness	72% (21)	67% (24)
Real word sentence completion^**^	64% (12)	53% (7)
Pseudo-word sentence completion^*^	60% (17)	54% (21)
Pseudo-word reading aloud	65% (18)	63% (18)

* p_2_ < 0.05;

** *p_1, 2_ < 0.001*.

As seen in Table 5, the native Hebrew speakers named significantly more objects correctly than did the language minority children [*t*_(110)_ = 6.155, *p* < 0.001, *d* = 1.162], as expected from previous studies (e.g., Bialystok et al., 2010; Farnia and Geva, 2011; Kieffer and Lesaux, 2012b). However, no group differences were found in phonological awareness performance [*t*_(110)_ = 1.353, *p* = 0.179], again as documented in previous studies (e.g., Geva and Yaghoub Zadeh, 2006; Kieffer and Vukovic, 2013).

Regarding morphological knowledge, no group differences were found in the task of pseudo-word reading aloud, which measures morphological processing and was not correlated with vocabulary knowledge. Both native Hebrew speaking students and language minority students assimilated the unvowelized pseudo-words to existing morphological patterns to the same degree [*t*_1(108)_ = 0.553, *p* = 0.581; *t*_2(19)_ = 0.719, *p* = 0.481]. This reading pattern conforms to the findings reported by Bar-On and Ravid (2011) using the same task among 4th grade native Hebrew speaking students.

In the pseudo-word sentence completion task, which measures morphological awareness, the difference between the native Hebrew speaking students and language minority students was marginal in the subject analysis [*t*_1(108)_ = 1.691, *p* = 0.094], and significant in the item analysis [*t*_2(13)_ = 2.922, *p* = 0.012], suggesting a difference between the groups. Although the experimental items in this task were non-words, accurate performance did recruit existing lexical and linguistic knowledge, because the targets were embedded in real sentences. Further, as reported above, this task was moderately correlated with vocabulary knowledge, again suggesting that it does rely to some extent on existing linguistic knowledge, thus leading to the observed difference in performance between the native Hebrew and the language minority students. Notably, the group difference was significant only in the item, and not in the subject analysis, suggesting that the effect is less robust in this measure than in the real-word sentence completion task.

Finally, in the real word sentence completion task, the native Hebrew speaking students received significantly higher scores than the language minority students in both the subject [*t*_1(110)_ = 3.74, *p* < 0.001, *d* = 1.12] and the item [*t*_2(27)_ = 2.549, *p* = 0.017] analyses. Thus, language minority students showed lower levels of performance when required to extract and manipulate morphological information from real words in Hebrew, putatively showing reduced morphological awareness.

**Figure 1:**
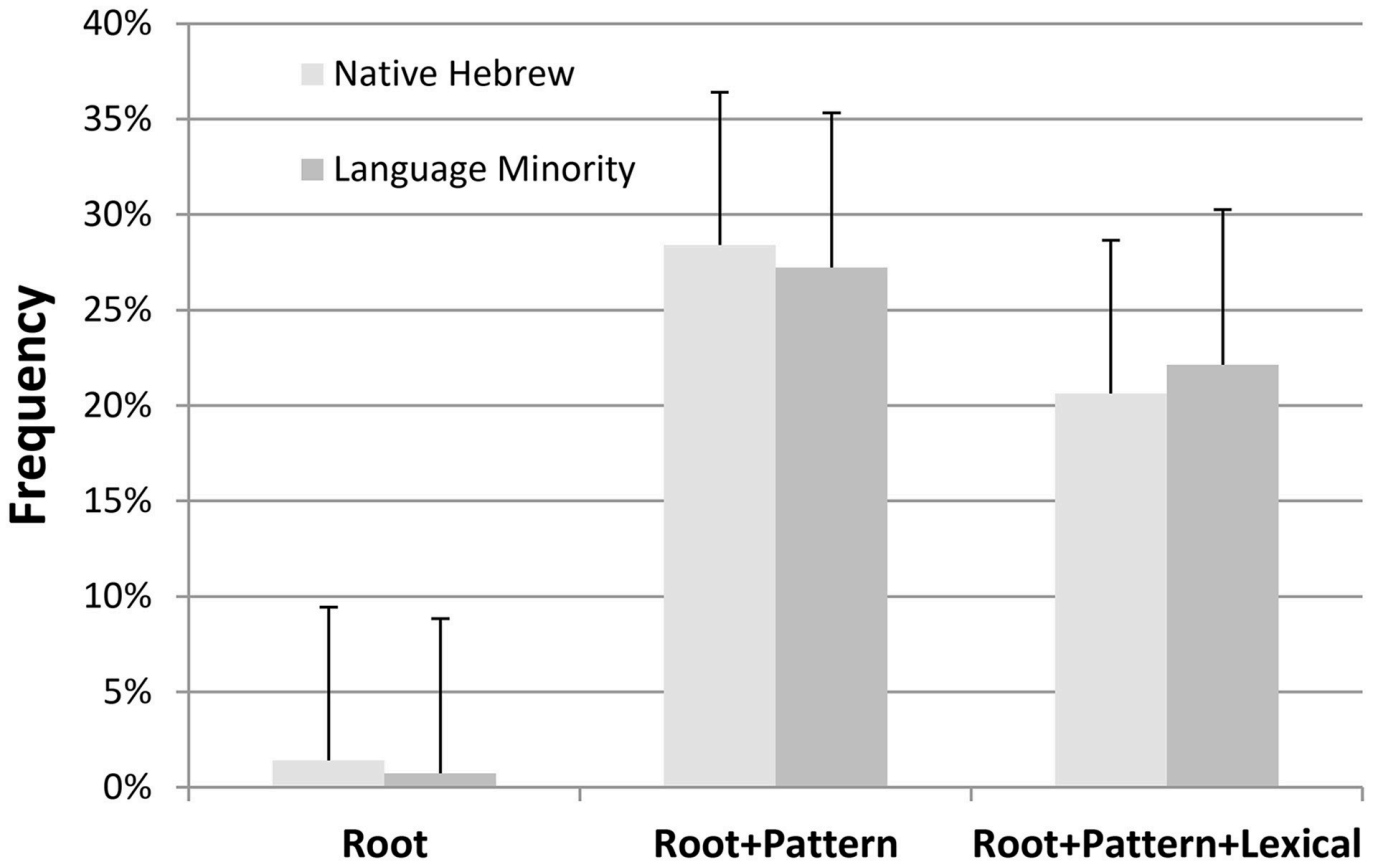
Frequency of error types (SEM) in the real word sentence completion task, by group.

However, an analysis of errors using the partial knowledge score demonstrated that although language minority students made more errors over all, there were no significant qualitative differences between participant groups in the type of errors they made (all *p* > 0.32). As presented in Figure 1, the frequencies of the different error categories were similar across groups.

In both groups, almost 50% of the errors were irrelevant answers or omitted responses (not depicted in Figure 1). The least frequent error (labeled in the graph as “Root”) was using a correct root in a syntactically inappropriate pattern. However, half of the errors in both groups were characterized by using general abstract morphological representations in order to overcome the lack of specific lexical knowledge. The most frequent error (labeled as “Root+Pattern”) in both groups was using the correct root in a syntactically appropriate morphological pattern, but the resulting response provided by the child was not an actual word in the Hebrew lexicon. For example, the correct response to completing the sentence “Dana **sawed** the board and now the board _____ (is sawed)” is *menusar*, derived by embedding the root *n.s.r* (carrying the meaning of sawing) in the specific adjectival pattern, meCuCaC. Some children, however, who putatively are not familiar with this lexical item, instead provided the non-word *nasur*, based on the correct root (*n.s.r*), again embedded in a different existing adjectival pattern CaCuC. Crucially, the resulting response *nasur* is not an actual word in Hebrew.

Finally, of similar frequency were the errors (labeled as Root+Pattern+Lexical) that reflected more developed lexical knowledge—such that the response provided by the child used the appropriate root embedded in a syntactically appropriate pattern resulting in a real word in Hebrew. However, in these cases the response was semantically inaccurate. For example, the correct response for completing the sentence “At first the orange was unripe and now it is **ripe**. What happened to the orange? The orange ____” is *hivSil* (ripened), based on the root *b.S.l* in the passive verbal form hiCCiC. However, many children provided the word *hitbaSel*, which uses the same root (*b.S.l*), embedded in a different verb pattern (hitCaCeC), and is thus morphologically appropriate. Yet, the meaning of the form *hitbaSel* is “was cooked (reflexive form),” and thus does not fit the sentence frame.

In the latter two error types, all children tended to use the correct root and an existing morphological pattern in an appropriate syntactic context and therefore both participant groups demonstrated equally developed abstract morphological awareness. Thus, the error analysis shows that both the language minority and the Hebrew native speaking children used their morphological awareness of roots and patterns in a similar manner in order to produce their responses, when they were not familiar with the specific lexical item required.

To summarize, we found that the different morphological measures were associated to different degrees with vocabulary knowledge, and this degree of association allows us to understand the pattern of group differences. Specifically, language minority students showed lower performance than that of native Hebrew students in the morphological task that correlated strongly with vocabulary knowledge. In the morphological task moderately correlated with vocabulary, language minority students showed marginally lower performance. Finally, in the morphological task that was uncorrelated with vocabulary, we found equivalent performance of the two groups.

### Morphological priming task

Correct RTs in the three experimental conditions (phonological control, pure morphologically related, morpho-semantically related) were averaged across subjects and across items, for the RT analyses. Accuracy rates were similarly averaged across subjects and across items (Table 6). Response latencies that deviated from each participant's mean in each condition by more than 2.5 standard deviations were eliminated from the RT analyses, (~2% of the data).

**Table 6:** Average RTs in milliseconds (*SD*), and accuracy rates (%) in the cross-modal priming task, by group.

	**Native Hebrew *N* = 56**		**Language minority *N* = 56**	
	**RT**	**ACC**	**RT**	**ACC**
Phonological control	1,477 (577)	86% (12)	1,727 (888)	79% (18)
Morphologically related	1,342 (537)	90% (12)	1,571 (931)	83% (16)
Morpho-semantically related	1,322 (481)	91% (9)	1,434 (645)	88% (11)
Morphological priming effect	135 (335)	3.5% (12)	156 (598)	4% (15)
Morpho-semantic priming effect	156 (435)	5% (11)	293 (538)	9% (17)

Task performance was analyzed using a two-way repeated measures ANOVA, with group as a between participants factor (Native Hebrew, Language Minority) and priming condition as a within participant factor (Phonological Control, Morphological prime, Morpho-semantic prime), and the parallel analysis over items. The main effect of priming condition was significant [*F*_1(2, 220)_ = 12.48, *p* < 0.001, $\eta_{p}^{2}$ = 0.102; *F*_2__(2, 92)_ = 4.16, *p* = 0.019, $\eta_{p}^{2}$ = 0.08]. The main effect of language group was not significant in the subject analysis (*p*_1_ = 0.107) but native Hebrew speakers were found to be significantly faster than language minority students in the item analysis [*F*_2__(1, 46)_ = 5.82, *p* = 0.02, $\eta_{p}^{2}$ = 0.112]. The two-way interaction was not significant in either analysis (*p*_1_ = 0.267, *p*_2_ = 0.217).

Planned comparisons showed that both the pure morphological priming effect and the morpho-semantic priming effect were significant (differed from zero, both *p* < 0.002). Further, the effects were equivalent for the two participant groups (both *p* > 0.139).

The analysis of accuracy paralleled that of RTs. The main effect of priming condition was significant [*F*_1(2, 220)_ = 15.418, *p* < 0.001, $\eta_{p}^{2}$ = 0.123; *F*_2__(2, 92)_ = 13.702, *p* < 0.001, $\eta_{p}^{2}$ = 0.23]. The main effect of language group was also significant [*F*_1(1, 110)_ = 7.348, *p* = 0.008, $\eta_{p}^{2}$ = 0.63; *F*_2__(1, 46)_ = 16.91, *p* < 0.001, $\eta_{p}^{2}$ = 0.269] because native Hebrew speakers were more accurate overall than language minority students. The two-way interaction was not significant in either analysis (*p*_1_ = 0.146, *p*_2_ = 0.256). Planned comparisons showed that both the pure morphological priming effect and the morpho-semantic priming effect were significant (differed from zero, both *p* < 0.003). Further, the effects were equivalent for the two participant groups (both *p* > 0.09).

As described above, the morphological priming task did not correlate with the other morphological tasks in the current study, suggesting that it taps additional processes such as decision making and speed of processing. Nonetheless, we replicate previous finding of significant morphological facilitation in this task, even in the absence of semantic relatedness (Frost et al., 1997, 2000). Somewhat surprisingly, the morphological priming effect was also non-correlated with vocabulary knowledge although the stimuli in the task were real lexical items. It seems that morphological facilitation was driven by participants' ability to extract the root of the word, which in Hebrew can be achieved without knowledge of the word meaning itself (Deutsch et al., 1998; Berent et al., 2004, 2007). Consistent with this argument, we found equivalent priming effects for the language minority and the native Hebrew speaking children, suggesting that this facilitation in processing is less reliant on existing lexical knowledge.

## Discussion

The current study explored the relations between lexical and morphological knowledge in school-aged children from two different perspectives. First, we investigated the links between lexical and morphological knowledge by using various tasks of morphological awareness and processing. Second, to investigate the possible differential impact of reduced language exposure, we compared the vocabulary and morphological knowledge of language minority children to that of their native speaking peers.

Our results show that although vocabulary and morphology are positively related, and both are driven by the amount of linguistic input, these two construct are separable, can be measured independently and their acquisition at least partially relies on distinct learning mechanisms. Specifically, the current findings indicate that the degree to which a specific measure of morphology recruited lexical knowledge determined the strength of its correlation with vocabulary. Thus, the morphological measures (with the exception of the priming task, which will be discussed separately) that used pseudo-words showed weaker associations with vocabulary. Second, the reduced exposure of language minority students to Hebrew led to reduced vocabulary knowledge. Nevertheless, at the same time, language minority children exhibited similar morphological knowledge to that of their native speaking peers, when abstract morphological patterns were measured. We argue that this abstract pattern learning is driven by type frequency exposure, which was sufficient in order to reach a “critical mass” and allowed both language groups to acquire abstract morphological knowledge. Before further discussing these two main findings, we first address the dimensionality of morphological knowledge.

### The complexity of measuring morphology

Based on previous research (Nagy et al., 2014; Tighe and Schatschneider, 2015; Goodwin et al., 2017), we originally conceived of the four morphological tasks included in the present study as being best described by a two-by-two structure along the dimensions of word/pseudo-word and awareness/processing (see Table 2). However, the current patterns of performance and cross-task associations suggest that this conception might not fully capture the complexity of morphological knowledge.

First, although three of the morphological tasks (real word sentence completion, pseudo-word sentence completion and pseudo-word reading) were significantly correlated with each other, the magnitude of morphological priming was not found to correlate with performance on the other morphological tasks. We have identified only a single previous study that combined a morphological priming task with an additional offline measure of morphological awareness (Kraut, 2015). However, in this study adult L2 learners of English failed to show a significant morphological priming effect and thus its putative relation with morphological awareness could not be examined.

A possible explanation for this difference between morphological priming and other measures of morphological knowledge might refer to the task demands. Specifically, the primed lexical decision paradigm is a speeded task that also includes a binary decision making process, neither of which is required in the other morphological tasks we employed, thus possibly leading to dissociations in performance. However, this is a tentative suggestion that should be fully explored in future research.

Second, the remaining morphological processing task (namely, pseudo-word reading aloud) correlated with the morphological awareness tasks as strongly as they correlated with each other. Therefore, at least in the current results, the awareness/processing dimension did not seem to capture important variability as originally conceived. In contrast, the dimension of word/pseudo-word stimuli, however, seems to offer the most informative description of the current tasks, but it too seems not to be binary, as we originally described. Specifically, it seems that not only the lexical status of the target stimuli themselves determines the degree to which a morphological task recruits lexical knowledge, but also the existence of a wider linguistic context.

### The relation of vocabulary and morphology

Previous research of school-aged children has established strong links between vocabulary and morphological knowledge (for native speaking children see McBride-Chang et al., 2008; Sparks and Deacon, 2015; for language minority children see Ramirez et al., 2011; Park et al., 2014), links that might at least be partially driven by the tools used to assess morphological knowledge. In the current study we replicate previously reported strong correlations between specific morphological tasks and vocabulary (McBride-Chang et al., 2005; Kieffer and Lesaux, 2012a; Spencer et al., 2015; Goodwin et al., 2017). Specifically, the real-word sentence completion task, which was the task of choice used in much previous research, was strongly correlated with vocabulary knowledge. In contrast, the pseudo-word reading aloud task did not correlate with vocabulary. However, the pseudo-word sentence completion task was moderately correlated with vocabulary (for similar results in adults, see Tighe and Schatschneider, 2015). As we suggest above, this patterns is most likely driven by the fact that although the targets themselves were pseudo-words, they were embedded in a meaningful sentence, the processing of which did recruit lexical knowledge.

A somewhat unexpected pattern was found in the priming task that used real word stimuli, as the pure morphological priming was not significantly correlated with vocabulary. We interpret this result in relation to the derivational structure of Hebrew, where the morphological root can be extracted without knowledge of the word meaning. Indeed, previous studies have demonstrated intact morphological processing of pseudo-words in Hebrew (Deutsch et al., 1998; Berent et al., 2004, 2007). Therefore, the degree of morphological facilitation was unrelated to children's lexical knowledge.

These findings, that not all morphological tasks were related to vocabulary knowledge, support the notion that the two constructs are separable, and can be measured independently (Tighe and Schatschneider, 2015). The strength of the relationship between lexical and morphological knowledge is dependent on the degree that a specific morphological measure recruited lexical knowledge.

### The differential impact of reduced exposure on morphology and vocabulary

The language minority students in the current study showed the well-documented impact of reduced exposure to the societal language on their productive vocabulary knowledge (Farnia and Geva, 2011; Mancilla-Martinez and Lesaux, 2011). Because language minority children distribute their language exposure between two languages, they accrue lower token frequency of words in each language and the result is a smaller vocabulary size in each language (Gollan et al., 2008; Bialystok et al., 2010).

However, the reduced exposure to Hebrew of language minority children was nonetheless sufficient in order to establish their knowledge of abstract derivational morphological structures, when these were tested independently from item-based knowledge. Specifically, language minority children exhibited significantly lower performance in the tasks most strongly correlated with vocabulary knowledge (real word sentence completion) and marginally lower performance in a task moderately correlated with vocabulary knowledge (pseudo-word sentence completion). However, they performed on par with native speaking peers in the two tasks uncorrelated with vocabulary knowledge (pseudo-word reading and morphological priming).

Finally, additional support for this notion comes from the error analysis in the real-word sentence completion task. Performance on the task itself was strongly correlated with vocabulary knowledge, and indeed language minority children showed lower performance. However, the error analysis revealed children's strategies exactly in those cases when they were unfamiliar with the appropriate lexical item. The pattern of errors was very similar across groups, demonstrating that both language minority and native Hebrew speaking children recruited abstract morphological knowledge regarding roots and patterns in a similar manner. Namely, all participants produced morphologically plausible responses to the same degree, again echoing the pattern evident across the other morphological tasks.

Thus, the current results demonstrate a differential impact of reduced exposure to the societal language on item specific vs. abstract pattern learning, again supporting the notion that the acquisition of these types of knowledge is at least partially separable. Whereas vocabulary knowledge is acquired almost exclusively through token exposure, morphological knowledge mostly relies on learners' ability to identify patterns based on generalizations arising from type frequency of similar items (Bybee, 1995; Clark, 1998; Tomasello, 2003; Lignos and Yang, 2018). Thus, language minority children accrue reduced exposure to specific lexical tokens, which was reflected in their lower lexical knowledge. At the same time, by the 5th grade, language minority children's exposure to the various derivational morphological types we examined had reached the “critical mass.” The current findings also align well with the previous findings that Russian language minority children acquired the regular forms of Hebrew plural inflections (type exposure) but were not familiar with all irregular plural forms (token exposure) (Schwartz et al., 2009, 2014). To conclude, language minority children had accrued the necessary amount of language type input needed for abstracting structural regularities (Marchman and Bates, 1994; Bybee, 1995; Tomasello, 2003; Gathercole, 2007), which enabled them to recruit abstract morphological knowledge in a manner similar to their Hebrew native speaking counterparts.

### Limitations and future research

As described above, the morphological priming task that we used did not correlate with the other morphological tasks, despite producing a robust morphological facilitation effect, therefore arguably measuring similar underlying abilities. Because this is the first study to our knowledge investigating this relation, it is difficult to know whether this result indeed reflects differences in task demands or might reflect low reliability of the morphological priming task itself. This issue should be directly and thoroughly addressed in future research.

In the current study, we did not measure the possible impact of transfer from L1 morphological knowledge on language minority children's knowledge of Hebrew morphology, though this factor has been investigated in some previous studies (e.g., Ramirez et al., 2011). Because of the high complexity of the current design, we decided not to include this aspect. This choice was further motivated by the great linguistic distance between children's L1 (Russian) and L2 (Hebrew) (Schwartz et al., 2009). Future research can further elaborate upon this aspect.

## Conclusions

The current study demonstrates from two different perspectives that although vocabulary and morphological knowledge are linked, and are driven by the same linguistic input, these constructs are separable and rely at least partially on different learning mechanisms. First, our results show that morphological tasks differ in the degree to which they rely on item-base lexical knowledge. These findings should be taken into consideration when interpreting previous reports of strong connections between lexical and morphological knowledge. Second, language minority students in the current study showed equivalent performance to their native speaking counterparts on measures of abstract morphological knowledge but lower performance on measures of morphological knowledge that were linked to lexical knowledge. This demonstrates that the language minority students had accrued a critical mass of type exposure, which supported the development of abstract morphological. This pattern of results again supports the separability of lexical and morphological knowledge.

Theoretically, our findings reinforce the distinction between token and type frequency, and their contributions to different aspects of language learning (Bybee, 1995; Tomasello, 2003; Ambridge et al., 2015). Practically, our results suggest that the assessment of morphological knowledge should be sensitive to the possible confound of vocabulary knowledge, especially in populations who speak more than one language, and divide their language exposure between them.

## Ethics statement

This study was carried out in accordance with the recommendations of “Data collection approval in educational institutes” by the Ministry of Education Chief Scientist Bureau with written informed consent from all subjects. All subjects gave written informed consent in accordance with the Declaration of Helsinki. The protocol was approved by the Ministry of Education Chief Scientist Bureau.

## Author contributions

DS-Y, ZE, and AP: Designed the study; DS-Y: Collected the data; DS-Y and AP: Analyzed results; DS-Y: Drafted the manuscript; DS-Y, ZE, and AP: Revised and approved the final version of the manuscript.

### Conflict of interest statement

The authors declare that the research was conducted in the absence of any commercial or financial relationships that could be construed as a potential conflict of interest.

## Footnotes

^1^Regarding Russian literacy of the language minority students, 37% reported no ability to read and write in Russian, 32% reported basic literacy skills and only 31% rated their Russian literacy skills as very good. Consistent with the educational policy in Israel, the public schools deliver literacy instruction only in Hebrew, so that any existing Russian literacy skills were taught either by family members or in afternoon classes.

^2^One item was removed from the original measure because the correct answer was ambiguous and in the pilot stage, no participant provided the expected answer.

^3^Full coding of partial answers was also calculated, as described by Bar-On and Ravid (2011), but lead to the same patterns as observed for the percent correct coding, and therefore is not further described here.

^4^In the initial analysis of the real-word sentence completion task, two items received very low accuracy across both participant groups (< 31%). Thus, we decided to eliminate them from further analyses. However, an analysis of the full set of items yielded the same overall patterns of results as reported for the corrected scale. Namely, there was a significant group difference in performance [*t*_(110)_ = 2.236, *p* = 0.025, *d* = 0.42], and the correlation with vocabulary knowledge was also highly significant (*r* = 0.56, *p* < 0.001).
