# Supplementary material for: Separability of Lexical and Morphological Knowledge: Evidence from Language Minority Children
Source: Front Psychol. 2018 Feb 21;9:163. doi: 10.3389/fpsyg.2018.00163 (PMC5826353; doi:10.3389/fpsyg.2018.00163)
Supplement: Supplementary file 1 [file DataSheet1.docx]

Appendix 1

Pseudo-word Sentence Completion (Pal II Hebrew version)

יש להשלים את המשפט ע"י בחירת אחת ממילות התפל (מילים שאינן אמיתיות אך נשמעות כמו מילים בשפה) כך שהמשפט ישמע תקין בעברית.

**דוגמא**

| - אחותי הצליחה ____ אֶת הספר. - אכלתי את ____ הטעימים. - שכנתי היא אישה ____. - פגשתי ____ יפות. | **לְהָפְקִיל** הִתְפַּקְלָה בָּמִפְקָל לְפִּקָלוֹן  פִּקְלוֹנוֹת **הַמּפְקָלִים** הִתְפַּקַלְתִּי בִּפְקִילוּת  **פַּקְלָנִית** הִתְפַּקְלָה פִּקוּל פַּקְלַנִיוֹת  פָּקַלְתִּי בִּפְקִילוּת פִּקוּל **פּוֹקְלוֹת** |
| --- | --- |

| 1. צבעתי את ארונות ____. | מְפוּקָלִים מִפְקָלוֹת הַמִּפְקָל בְּפָקֹל |
| --- | --- |
| 1. שכחתי היכן הנחתי את ____. | פּוֹקְלוֹתַי הִתְפַּקַלְתִּי מִפְקָלוֹת הִתְפַּקְלוּת |
| 1. ביקשתי מאחותי שתארוז את ____. | מַפְקִלֶיהַ מְפוּקָלִים פִּיקַלְתִּי תְפַקֵל |
| 1. האורחים התבקשו על ידי המארח ____. | מְפוּקָלִים הַמּפוּקָל הִתְפַּקֵל לְהִתְפַּקֵּל |
| 1. חברתי ____ היא ציירת מחוננת. | מִתְפַּקֶלֶת הוּפְקְלָה הַמַּפְקִילָה פָּקַלְתִּי |
| 1. הרופאה שאליה פנה המטופל ____ מאוד. | פִּיקֵל מְפוּקֶלֶת פַּקְלָן שֶׁפִּיקֵל |
| 1. האישה ____ את התיק עלתה לחדרה. | שֶׁפָּקְלָה הִפְקִילָה הַמְּפוּקֶלֶת שֶׁהוּפְקָל |
| 1. הנערה שאותה ____ הנער חייכנית. | לְפַלְקֵל מוּפְקָל מַפְקִילָה פּוֹקֵל |
| 1. לדעתי, העיר שֶׁבה ____ יפה. | הִפְקִילָה פָּקַלְנוּ פְּקוּלָה פְקִילוּת |
| 1. מתי ניסה סגנו של היועץ ____? | בִּפְקִילוּת הוּפְקָל לְפַקֵּל הַמְּפוּקַל |
| 1. המנהלת שכנעה את ____ המפעל להציג לה את עיקרי תוכניתן. | פּוֹקֶלֶת פּוֹקְלוֹת פּוֹקְלֵי פּוֹקֵל |
| 1. השומר ביקש מן ____ לפתוח בפניו את השקיות הגדולות שהחזיקה. | הַפּוֹקֶלֶת הַפּוֹקֵל הַפּוֹקְלוֹת הַפּוֹקְלִים |
| 1. המשכנו ללכת למרות ____ הגדוֹלה. | שֶׁהִתְפַּקַלְנוּ הַפּיקָלוֹן פְּקִילוּתֵנוּ הִתְפַּקְלוּת |
| 1. ____ מתוארות קורותיה של משפחתי. | הַפּוֹקְלוֹת הִפְקַלְתִּי פֹּוקְלוֹת בָּפֶּקֶל |

Appendix 2

Correlations among Morphological Knowledge, Vocabulary and Phonological Awareness Tasks, by Language Group

|  | **Native Hebrew Speakers** | | | | | **Language Minority** | | | | |
| --- | --- | --- | --- | --- | --- | --- | --- | --- | --- | --- |
|  | **2** | **3** | **4** | **5** | **6** | **2** | **3** | **4** | **5** | **6** |
| 1. Real Word Sentence Completion | .34 | .36* | -.11 | .61** | .46** | .56** | .25 | -.20 | .69** | .42** |
| 1. Pseudo-word Sentence Completion |  | .41** | -.14 | .31 | .42** |  | .44** | .03 | .52** | .49** |
| 1. Pseudo-word Reading Aloud |  |  | -.16 | .25 | .48** |  |  | .11 | .24 | .48** |
| 1. Morphological RT Priming Effect |  |  |  | -.12 | .02 |  |  |  | -.12 | .09 |
| 1. Productive Vocabulary |  |  |  |  | .37** |  |  |  |  | .24 |
| 1. Phonological Awareness |  |  |  |  |  |  |  |  |  |  |

*p < .01; **p < .005
